# Symptom Distress Associated with Biopsy in Women with Suspect Breast Lesions

**DOI:** 10.5402/2012/898327

**Published:** 2012-07-08

**Authors:** Jayesh Kamath, Dean G. Cruess, Kevin Claffey, Lori Wilson, Natalie Phoenix, Susan Tannenbaum

**Affiliations:** ^1^University Connecticut Health Center, 10 Talcott Notch Road, East Lobby, 3rd Floor, Farmington, CT 06030, USA; ^2^The Carol and Ray Neag Comprehensive Cancer center, University of Connecticut School of Medicine, Farmington, CT 06030, USA; ^3^Department of Psychology, University of Connecticut, Storrs, CT 06269, USA

## Abstract

*Purpose*. To investigate symptom distress, quality of life, affective states, and inflammatory biomarkers before and after breast biopsy in women undergoing breast biopsy. *Methods*. A convenience sample of 47 women undergoing breast biopsy was assessed at the pre- and post-biopsy visits. The assessments included evaluation of fatigue, anxiety, depression, sleep disturbances, positive and negative affect, quality of life using validated self report measures, and a blood draw to determine markers of inflammation. *Results*. At the postbiopsy visit, a total of 15 participants were diagnosed with breast cancer, and 32 participants received negative biopsy result. The mean anxiety and sleep disturbances scores were in the clinically significant range for the total sample and for the biopsy positive (BC+) and biopsy negative (BC−) subgroups at both time points. For both subgroups, anxiety and sleep disturbances scores did not change significantly from pre- to post-biopsy. A subpopulation of participants in both groups reported moderate-to-severe anxiety, depression and fatigue levels at both time points. The inflammatory markers did not show consistent associations with psychosocial symptoms. *Conclusions*. A subset of participants in BC+ and BC− subgroups experience heightened symptom distress and negative impact on quality of life at both pre- and post-biopsy time points.

## 1. Introduction

Widespread use of screening, along with treatment advances, has been credited with significantly reducing breast cancer mortality [1]. However, the screening and diagnostic process can also result in heightened distress, which for some women can persist from months to years with a significant negative impact on quality of life [2–4].

Several studies have documented psychological and symptom distress associated with the screening process and with false positive or inconclusive results [5–9]. For women with suspected breast lesions, the immediate next step after screening is the biopsy process, which includes prebiopsy surgical consultation, followed by a biopsy procedure and finally a discussion of biopsy results. This biopsy process can lead to higher levels of distress compared to screening due to its proximity to a potential breast cancer diagnosis [10–12]. Positive biopsy results can result in heightened anxiety and distress due to thoughts about cancer treatments and prognosis [9, 10]. It is possible that negative biopsy might not completely resolve psychological distress and the fear generated by the abnormal mammogram and subsequent biopsy process [13, 14]. Therefore, it is important to investigate distress associated with the biopsy process in women undergoing this process regardless of diagnostic outcomes.

Evidence suggests that among women undergoing breast cancer treatment, psychological distress is positively associated with adverse physical symptoms such as pain, fatigue, and nausea [15–17]. Studies conducted among patients during or even after completion of breast cancer treatments also suggest high rates of these symptoms [18–20]. In addition, evidence suggests significant association between these symptoms of psychological distress and impaired functioning [16, 17, 20, 21]. Emerging evidence also supports a common pathophysiology and biological substrates underlying these symptoms in breast cancer survivors [22, 23]. Specifically, higher levels of proinflammatory cytokines, such as interleukin-6 (IL-6), have been found in depressed and fatigued breast cancer survivors in comparison with controls without these symptoms [24, 25].

Investigation of psychological distress early on during the diagnostic process might help identify women at risk for chronic psychosocial issues later on during their cancer treatments. This in turn might help us identify specific risk/protective factors for such women and help devise focused interventions. A prior study has documented significant psychological distress, high rates of psychiatric syndromes, and impaired functioning in women at the time of prebiopsy surgical consultation [10], and others have conducted longitudinal evaluation of mood, anxiety and coping mechanisms before and after biopsy [11, 12]. To our knowledge, however, no study to date has conducted a comprehensive investigation of symptom distress (including fatigue and insomnia), affective state, quality of life, and biomarkers longitudinally during the biopsy process, irrespective of diagnostic outcomes.

In the present study, we investigated symptom distress, quality of life and affective states at two time points during the biopsy process, at the time of prebiopsy surgical consult and after biopsy, after participants have received either a positive or negative diagnosis. We hypothesized that individual distress symptoms (e.g., depression, anxiety, fatigue, and insomnia) would be positively related to each other and would have a negative impact on the quality of life and affective state of the participants. We also hypothesized that distress would be more greatly reduced at the postbiopsy time point in women receiving a negative diagnosis compared to women receiving a positive diagnosis. In exploratory analyses, specific markers of inflammation were examined to evaluate their association with distress symptoms under investigation.

## 2. Methods

### 2.1. Participants and Procedures

The study protocol and procedures were approved by the University of Connecticut Health Center (UCHC) Institutional Review Board. Potential participants were recruited from the Breast Clinic at The Carole and Ray Neag Comprehensive Cancer Center at the UCHC. Participants included a convenience sample accrued from a group of consecutive patients referred to the surgeons at the cancer center due an abnormal breast imaging study or palpable breast abnormality. Participants were approached and invited for study participation by the study staff during their initial surgical consult if biopsy was planned. Written consents were obtained prior to any study procedures. Study eligibility criteria were as follows: women 18 years of age or older, without a prior history of breast cancer, with an abnormal breast imaging study or a palpable breast mass in whom biopsy was recommended. Patients with history of nonbreast malignancies were eligible if they have been disease-free for 5 or more years prior to study enrollment and were deemed by their physician at low risk for recurrence.

**Figure 1:**
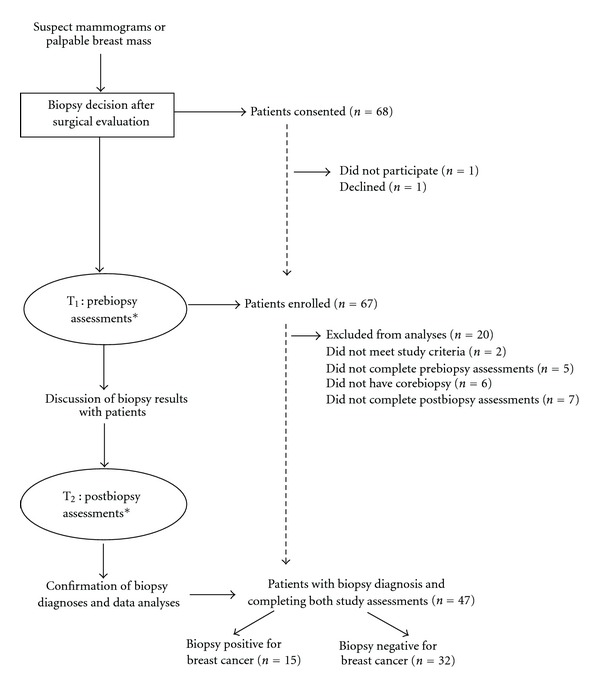
Study design and patient flow (*assessments: the questionnaires and blood draws).

Participants completed study assessments at two time points (Figure 1). The first set of assessments was conducted before biopsy (i.e., at the time of initial surgical consult); the second set of assessments was conducted after biopsy, after participants received results of their breast biopsy. Participants completed the questionnaires in the consult office within the cancer center, when possible, or, on occasion, from home if the patient could not stay to complete the measures. Both assessments included identical self-report questionnaires. Participants were also asked to provide blood samples for biological analyses at each time point. Participant's biopsy results were retrospectively obtained by the study staff at the time of data consolidation from the pathology reports and charts.

### 2.2. Measures

The measures included standardized demographic assessments, symptom questionnaires, and measures of quality of life and affective state. Fatigue was assessed using the Brief Fatigue Inventory (BFI). The BFI is a 9-item questionnaire for evaluation of fatigue in patients with cancer [26]. It is designed to evaluate severity and impact of fatigue on daily functioning in the past 24 hours and has been validated in patients with cancer [27]. The questions are based on a scale of 0–10, and the scores are averaged to provide a total BFI score. Higher scores on BFI suggest higher level of fatigue. The Hospital Anxiety and Depression Scale (HADS) was used to evaluate depressive and anxiety symptoms [28]. The HADS is a 14-item questionnaire consisting of two separate subscales, each consisting of 7 items, for anxiety (HADS-A) and depression (HADS-D). It is frequently used to evaluate depression and anxiety symptoms in patients with concurrent physical illness as it focuses on psychological symptoms [29] and has been validated in patients with cancer [30]. Higher total scores on HADS as well as higher scores on subscales suggest greater severity of symptoms. The Pittsburgh Sleep Quality Index (PSQI) was used to assess sleep dysfunction [31]. This scale has demonstrated internal consistency, convergent and discriminant construct validity and consistent reliability, across multiple samples, included in patients with breast cancer [32]. A total of 19 items in this scale provide 7 component scores, each ranging from 0–3 for a maximum global sum of 21. Higher scores on global index as well as component scales of the PSQI represent poorer sleep quality. Quality of life was evaluated using the European Organization for Research and Treatment of Cancer Quality of Life Questionnaire (EORTC QLQ-C30) [33, 34]. The EORTC QLQ-30 is a cancer-specific questionnaire that addresses various domains of quality of life [35]. It contains five function subscales, three symptom subscales, and two single items assessing global health and overall quality of life, and a number of single items addressing various symptoms and perceived financial impact [35]. Higher total score on EORTC represents better quality of life. Affective state of participants was assessed using the Positive and Negative Affect Schedule (PANAS) [36]. The PANAS is a 20-item self-report measure that consists of two 10-item mood scales, representing positive affect (PA) and negative affect (NA). Each item is rated on a 5-point Likert scale. The PANAS has shown high internal consistency and discriminant and convergent validity [36, 37]. Higher scores on PANAS subscales represent greater positive and negative affects. The Modified Social Support Survey (MSSS) is a modified version of the Social Support Survey developed as part of the Medical Outcomes Study in order to assess perceived social support [38]. The total MSSS score ranges from 18 to 90 with higher scores indicating greater perceived support. The MSSS has shown good convergent and discriminant validity in patients with medical illness [38].

### 2.3. Biological Assays

Two inflammatory markers, C-reactive protein (CRP), and interleukin-6 (IL-6), were assayed at both time points during the study. For this purpose, blood samples were collected from participants via inner elbow venipuncture and collected into vacutainer tubes. Samples were prepared via centrifuge, and serum was collected and subsequently frozen at −80°C. Serum was then analyzed for levels of CRP and IL-6 using Luminex Multiplex Bead assays according to manufacturer's instructions [39].

### 2.4. Data Analyses and Statistical Plan

All data analyses were performed using SPSS version 17.0 (SPSS, Inc., 2009). Means and standard deviations were computed to describe the sample and *t*-tests were used to compare the severity of symptoms at the prebiopsy and postbiopsy visits, as well as to compare the cancer positive and cancer negative groups at the postbiopsy visit. Patient subgroups were divided and compared at both visits for the total sample (regardless of diagnosis) and for breast cancer positive (BC+) and breast cancer negative (BC−) subgroups separately, and severity of symptoms was evaluated based on established cutoffs for depression, anxiety, and fatigue questionnaires. Pearson correlations were computed to evaluate relationships between symptoms (anxiety, depression, sleep disturbances and fatigue), affective state, and quality of life at both time points. Partial correlations were computed in order to allow for determination of associations between all study measures with appropriate covariates included. Potential covariates included participant's age, time interval between pre- and post-biopsy time points as well as several other pertinent demographic/medical variables.

To ensure normal distribution of scores, values above or below three standard deviations of the mean for CRP and IL-6 were removed for further analyses. Square root and natural log transformations were then conducted for CRP and IL-6, respectively. Bivariate correlations were computed in order to determine associations between the behavioral measures and biological markers (CRP and IL-6) and between these variables and potential covariates. Finally, residualized change scores were computed to further examine relationships between study measures over time controlling for baseline values.

## 3. Results

### 3.1. Patient Characteristics

A total of 47 patients completed both study assessments at pre- and post-biopsy visits and had a confirmed biopsy result. At the postbiopsy visit, 15 participants (31.9%) were diagnosed with breast cancer, and 32 participants (68.1%) received a negative biopsy. The average age of participants was 52.93 years (SD = 10.67); 56.6 years (SD = 8.93) for participants with positive biopsy; 51.22 years (SD = 11.11) for participants with negative biopsy results. Notably, higher number of participants (43%) in the positive biopsy group were 60 years of age or older, and this group had no participants under the age of 45. Comparatively, only 18% of participants in the negative biopsy group were above 60 years of age and 31% of participants in this group were under 45 years of age. Participants were mostly Caucasian (51.1%) with smaller numbers of African-American (4.3%) and Hispanic (6.4%) women, with similar numbers in both biopsy positive and biopsy negative subgroups. Information on ethnicity was not available for 18 of 47 participants. The mean interval of time between completion of the two sets of study questionnaires was 25.5 days (SD = 26).

### 3.2. Descriptive Statistics of Entire Sample and Patient Subpopulations

The descriptive statistics for all study outcome measures for both breast cancer positive (BC+) and breast cancer negative (BC−) are displayed in Table 1. The mean anxiety and sleep disturbance scores were in clinically significant range at both time points for the total sample and for both patient subgroups. Notably, anxiety scores did not change significantly from pre- to post-biopsy for either the BC+ (*t* (14) = −.202, *P* = .843) or the BC− (*t* (31) = 1.067, *P* = .294) subgroup. Similarly, sleep disturbances also remained unchanged from pre to post-biopsy for both the BC+ (*t* (13) = .915, *P* = .377) and BC− (*t* (31) = .293, *P* = .771) subgroups. The mean scores for depressive and fatigue symptoms were in the normal ranges as defined for these measures [26, 29] at both time points for the total sample as well as for both patient subgroups. To assess scores on the PANAS, we used as a comparison sample data from a large (1,003) general population sample of adult men and women [37]. Results indicated that positive and negative affects for the total sample and subgroups were within normal range at both pre- and post-biopsy time points. There was no significant change in IL-6 across time for the total sample, or for the BC+ or BC− subgroups. Repeated measures analyses did indicate a significant main effect for CRP, with levels of CRP increasing from pre- to post-biopsy time points for both subgroups (*F* (1, 41) = 4.241, *P*≤.05).

**Figure 2:**
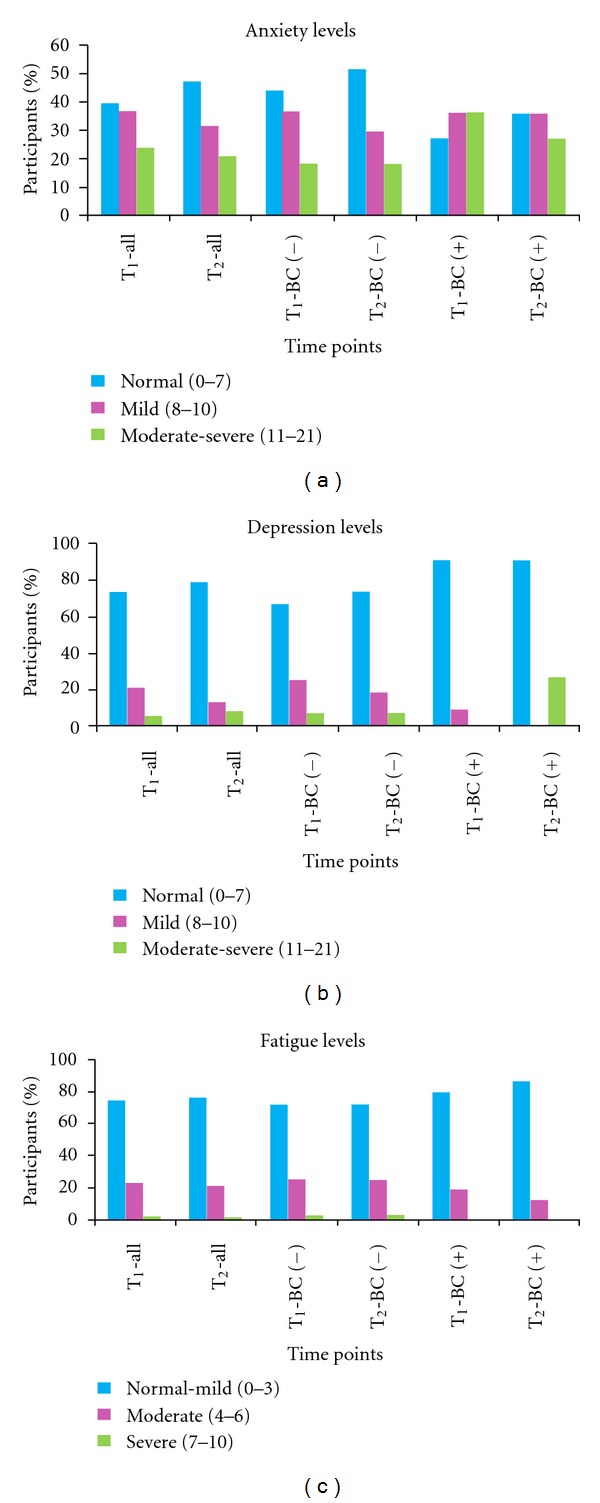
Anxiety, depressive and fatigue symptoms for all and subgroups of participants at prebiopsy (T_1_) and postbiopsy (T_2_) time points and (all-all participants, BC (+): breast cancer positive, BC (−): breast cancer negative).

Participants were divided in three subgroups based on established cutoffs for the respective scales [26, 29] to identify patients experiencing mild, moderate, or severe symptoms (Figure 2). Our objective was to assess how these patient subpopulations changed from pre- to post-biopsy time points. For the BC− subgroup, the percentage of patients reporting moderate-to-severe anxiety symptoms did not change from pre-to post-biopsy time points despite negative biopsy results. In the BC+ subgroup, the percentage of patients reporting moderate to severe symptoms reduced from pre-(*T*_1_: 36.4%) to post-biopsy (*T*_2_: 27.3%) time points. The subgroup of patients reporting moderate to severe level of fatigue at both time points was higher in the BC− subgroup (*T*_1_: 28.1% and *T*_2_: 28.1%) than the BC+ subgroup (*T*_1_: 20% and *T*_2_: 13.3%). None of the differences or changes in percentages were statistically significant. Analyses of severity of depressive symptoms indicated that most patients remained in the normal-to-mild range at both time points.

### 3.3. Psychological Distress Associations

Correlational analyses using mean scores (Table 2) were conducted to investigate if the symptoms correlated with each other, with affective states, were modified by perceived social support and had an impact on quality of life. For the total sample, the analyses across both time points indicated that anxiety symptoms were significantly related to fatigue, depression, and sleep impairment and had a negative impact on quality of life as measured by the EORTC. Anxiety was significantly related to negative affect and inversely correlated with positive affect. Similar to anxiety, fatigue significantly correlated with every other measured symptom and had a significant negative impact on quality of life. Additionally, fatigue had a significant positive correlation with negative affect and significant negative correlation with positive affect. All of the evaluated symptoms showed a negative impact on quality of life. Quality of life was significantly related to negative affect and was inversely related to positive affect. Perception of social support was positively correlated with positive affect and was inversely related to sleep disturbances and depression.

We also conducted regression analyses to examine within subjects, between subjects, and interaction effects for time point and diagnostic status across all variables of interest. For all of the assessed symptoms, that is, anxiety, depression, sleep, and fatigue, the effects of time (i.e., pre- versus post-biopsy) or of cancer status or their interactions was not significant (*P'*s >.10). Similarly the effect of time and cancer status as well as their interactions was not significant for affective states of the participants (*P'*s >.10).

### 3.4. Distress/Inflammation Marker Associations

The markers of inflammation (CRP and IL-6) did not show consistent correlations with specific symptoms or affective states. However, we did find sporadic correlations. For example, BC− patients were found to have a positive correlation with IL-6 and level of depression (*P* < 0.01). On the other hand, BC+ patients were found to have a negative correlation with IL-6 and quality of life (*P* < 0.01) and a positive correlation between CRP and level of fatigue (*P* < 0.05). For the total sample, regardless of diagnosis, IL-6 correlated to level of depression (*P* < 0.01).

## 4. Discussion

The overarching purpose of the present study was to investigate symptom distress and psychological impact of the biopsy process on women prior to and following breast biopsy. Overall, the majority of patients in both BC+ and BC− groups reported minimal symptom distress and minimal impact on quality of life. These results are consistent with past studies utilizing similar longitudinal assessments during the biopsy phase [11, 12]. These results diverge from a cross-sectional study in which the authors found higher levels of psychological distress and psychiatric symptomatology [10]. The difference in these two studies can be found in the timing of assessments and population. The assessments in that study [10] were conducted at the time of surgical consultation and only in BC+ patients; whereas the assessments in the present study were conducted before and after biopsy in both BC− and BC+ patients. This difference in methodology may have accounted for the disparate findings.

In the present study, most patients did not experience clinically significant levels of fatigue or depressive symptoms, and almost half of the total participants did not experience clinically significant anxiety symptoms during the biopsy process. However, as expected, clinically significant anxiety and sleep impairment in a significant subpopulation of patients were found. Notably, these symptoms remained elevated at the post-biopsy visit, even in patients receiving a benign diagnosis. This suggests that a subgroup of patients experience heightened distress and sleep problems during the biopsy process, irrespective of diagnostic outcomes. It is possible this some of these women had elevated distress prior to their mammograms. A small subpopulation of patients reported moderate levels of fatigue at both time points. Fatigue has been identified as the most common symptom in patients with breast cancer during and years after cancer treatments [40, 41]. Identification of fatigue in a subset of patients at the diagnostic stage, prior to any cancer treatments, is an important finding and warrants further investigation. Fatigue in this patient population was associated with anxiety, depression, sleep impairment, negative affect and was inversely related to positive affect. This implies potential contribution of psychological mechanisms in the pathophysiology of fatigue.

The study findings suggest that individual vulnerability to distress might be a crucial factor determining psychological and physical impact on the biopsy process. Several findings support this conclusion as follows. The time points (pre- versus post-biopsy) and more importantly, cancer status, did not have a significant impact on any of the distress symptoms under investigation. Additionally, several of the symptoms under investigation correlated with each other in both subgroups. Also, to our surprise, certain symptoms such as percentage of patients experiencing moderate-severe fatigue was higher in the BC− subgroup compared to the BC+ subgroup at both time points.

The study results are partially consistent with other prior work that has found women with specific personality factors, prebiopsy high levels of anxiety, and negative coping mechanisms (such as cognitive avoidance coping) had high level of distress and anxiety after biopsy [11, 12]. Our findings are also consistent with findings from a recent, cross-sectional study evaluating distress in women with benign breast biopsy [42]. Approximately one-third of women in that study reported the biopsy process “very distressing.” Our findings seem to somewhat diverge from past findings at our second, postbiopsy time point. In past studies, the BC− group seems to show improvement in emotional distress from pre-to post-biopsy in comparison with the BC+ group [11, 12], but participants in our study do not. There might be several reasons for these seemingly differential findings. First, we had specific, individual measures for symptoms, while past studies included an overall measure of mood state, that is, (POMS). One might argue that that the symptom-specific scales in the present study provided a better, more comprehensive assessment of symptoms than the POMS scale, at least in the subgroup of women with heightened distress.

Fatigue and depression in patients undergoing breast cancer treatments or in breast cancer survivors has been associated with chronic inflammation [23–25]. In the present study, no consistent and clinically significant correlations of inflammation markers with symptoms under investigation were found. This might be due to the small sample size or might be due to a possibility that no such associations exist at the time of breast cancer screening/diagnostic workup. It is possible that any increase in inflammation with associations to specific psychological symptoms might evolve over time as a consequence of cancer treatments and play a role in the pathophysiology of persistent physical/psychological distress.

There are several limitations of the present study, including a relatively small sample size and convenience sample. Collection of demographic and clinical assessments was also limited in an effort to reduce patient burden. Comprehensive assessment of these factors [42] could have identified specific risk factors for distress during the biopsy process. Such an assessment with longitudinal assessment of symptom distress could have helped stratify the distress risk during the biopsy process. More psychosocial assessment at baseline with collections of specific past information (e.g., psychiatric history, family psychiatric history as well as predisposing factors for breast cancer like strong family history) could provide a better picture of environmental stressors as well as vulnerability to psychological distress in this setting and may have further helped to understand some of the current findings.

The present study identified a subset of women with heightened distress despite negative biopsy results. Future work should attempt to identify these vulnerable women prior to mammograms and consider development of specific distress management strategies for these women at this emotionally stressful time. Future projects should also attempt to monitor this subset of women, both from a psychological standpoint and to ensure their compliance with monitoring recommendations. Limited sample size of the present study restricted further analyses of the impact of age difference between BC+ and BC− subgroups. Future studies should investigate the impact of this critical variable [43] on psychological distress during the biopsy process.

## 5. Conclusions

To our knowledge, the present study is the first study of its kind with longitudinal and comprehensive evaluation of symptom distress, affective state, and quality of life in women undergoing the biopsy phase of breast cancer diagnosis. The study results suggest that majority of patients continue to farewell even after receiving a positive diagnosis. However, a subset of patients, irrespective of diagnostic status, experience heightened symptom distress with negative impact on their quality of life. Thus, psychosocial interventions may be warranted early on, especially in this subset of patients, to address psychological and symptom distress and improve quality of life.

## Figures and Tables

**Table 1:** Descriptive statistics for outcome measures for ALL participants and by diagnostic status^∗^ at pre- (*T*_1_) and post-biopsy (*T*_2_) visits.

Outcome measure	Visit	All participants	BC positive participants	BC negative participants
		Mean ± SD	Mean ± SD	Mean ± SD
Anxiety subscale of HADS	*T*_1_	8.53 ± 3.63 (*n* = 38)	9.00 ± 2.37 (*n* = 11)	8.33 ± 4.06 (*n* = 27)
	*T*_2_	7.61 ± 3.49 (*n* = 38)	8.46 ± 2.88 (*n* = 11)	7.26 ± 3.71 (*n* = 27)
Depressive subscale of HADS	*T*_1_	4.66 ± 3.57 (*n* = 38)	3.91 ± 3.08 (*n* = 11)	4.96 ± 3.77 (*n* = 27)
	*T*_2_	4.08 ± 4.05 (*n* = 38)	3.45 ± 3.59 (*n* = 11)	4.33 ± 4.26 (*n* = 27)
Fatigue (BFI)	*T*_1_	2.78 ± 2.17 (*n* = 47)	2.79 ± 1.34 (*n* = 15)	2.77 ± 2.49 (*n* = 32)
	*T*_2_	2.68 ± 1.99 (*n* = 47)	2.62 ± 1.34 (*n* = 15)	2.70 ± 2.25 (*n* = 32)
Fatigue level (BFI)	*T*_1_	1.28 ± 0.50 (*n* = 47)	1.20 ± 0.41 (*n* = 15)	1.31 ± 0.54 (*n* = 32)
	*T*_2_	1.26 ± 0.49 (*n* = 47)	1.13 ± 0.35 (*n* = 15)	1.31 ± 0.54 (*n* = 32)
Sleep (PSQI)	*T*_1_	7.13 ± 3.59 (*n* = 47)	6.60 ± 2.64 (*n* = 15)	7.38 ± 3.97 (*n* = 32)
	*T*_2_	7.15 ± 3.72 (*n* = 46)	6.86 ± 3.94 (*n* = 14)	7.28 ± 3.67 (*n* = 32)
PANAS positive subscale	*T*_1_	33.17 ± 9.58 (*n* = 47)	33.60 ± 6.48 (*n* = 15)	32.97 ± 10.83 (*n* = 32)
	*T*_2_	33.39 ± 9.30 (*n* = 46)	31.57 ± 7.17 (*n* = 14)	34.19 ± 10.09 (*n* = 32)
PANAS negative subscale	*T*_1_	21.72 ± 7.75 (*n* = 47)	21.73 ± 7.75 (*n* = 15)	21.72 ± 7.88 (*n* = 32)
	*T*_2_	20.83 ± 7.84 (*n* = 46)	23.21 ± 8.41 (*n* = 14)	19.78 ± 7.48 (*n* = 32)
Quality of life (EORTC)	*T*_1_	74.82 ± 18.91 (*n* = 47)	77.78 ± 12.86 (*n* = 15)	73.44 ± 21.21 (*n* = 32)
	*T*_2_	71.28 ± 21.69 (*n* = 47)	69.44 ± 15.00 (*n* = 15)	72.14 ± 24.37 (*n* = 32)
Modified social support (MSSS)	*T*_1_	76.56 ± 22.63 (*n* = 46)	79.58 ± 17.63 (*n* = 14)	75.24 ± 24.64 (*n* = 32)
	*T*_2_	77.52 ± 26.81 (*n* = 47)	89.57 ± 16.77 (*n* = 15)	71.88 ± 28.92 (*n* = 32)
C-reactive protein (CRP)^∗∗^	*T*_1_	7248.06 ± 7823.91 (*n* = 47)	6601.80 ± 8583.84 (*n* = 15)	7551.00 ± 7567.07 (*n* = 32)
	*T*_2_	8358.26 ± 8597.71 (*n* = 47)	9696.07 ± 11426.52 (*n* = 15)	7731.16 ± 7032.20 (n=32)
Interleukin-6 (IL-6)^∗∗^	*T*_1_	348.59 ± 616.14 (*n* = 34)	273.84 ± 472.48 (*n* = 11)	384.34 ± 681.06 (*n* = 23)
	*T*_2_	296.29 ± 556.48 (*n* = 39)	213.49 ± 443.79 (*n* = 14)	342.66 ± 614.32 (*n* = 25)

BC: breast cancer, ^∗^Diagnostic status (BC+ or BC−) was determined after biopsy. It is important to note that participants are not aware of their BC status at the first (*T*_1_) visit, ^∗∗^Marker on inflammation.

**Table 2:** Associations between measures of distress, affective state, quality of life, and perceived support^*∧*^.

	PANAS positive	PANAS negative	PSQI	Anxiety	Depression	QOL	Fatigue	Social Support
				(HADS-A)	(HADS-D)	(EORTC)	(BFI)	(MSSS)
PANAS positive	1							
PANAS negative	*R *=* −*.125	1
	*P *=.232	
Sleep (PSQI)	*R *=* −*.167	*R *=.248	1
	*P *=.112	*P *=.017^∗^	
Anxiety (HADS-A)	*R *=* −*.192	*R *=.564	*R *=.368	1
	*P *=.098^†^	*P < *.001^∗^	*P *=.001^∗^	
Depression (HADS-D)	*R *=* −*.556	*R *=.171	*R *=.048	*R *=.246	1
	*P *=.001^∗^	*P *=.143	*P *=.679	*P *=.032^∗^	
QOL (EORTC)	*R *=.552	*R *=−.330	*R *=−.308	*R *=* −*.208	*R *=* −*.459	1
	*P *=.001^∗^	*P *=.001^∗^	*P *=.003^∗^	*P *=.072^†^	*P *=.001^∗^	
Fatigue (BFI)	*R *=* −.*307	*R *=.365	*R *=.297	*R *=.396	*R *=.367	*R *= *−*.564	1
	*P *=.003^∗^	*P < *.001^∗^	*P *=.004^∗^	*P < *.001^∗^	*P *=.001^∗^	*P < *.001^∗^	
Social support (MSSS)	*R *=.266	*R *=.047	*R *=−.233	*R *=−.067	*R *=−.198	*R *=.122	*R *=* −*.085	1
	*P *=.010^∗^	*P *=.659	*P *=.026^∗^	*P *=.565	*P *=.088^†^	*P *=.244	*P *=.466	

^*∧*^For the total sample and averaging scores across both time points, ^∗^Significant (*P* < 0.05), ^†^Trend toward significance (0.10 > *P* ≥ 0.05), PANAS: Positive and Negative Affect Scale, PSQI: Pittsburgh Sleep Quality Index, HADS: Hospital Anxiety and Depression Scale, BFI: Brief Fatigue Inventory, EORTC: European Organization for Research and Treatment of Cancer, MSSS: Modified Social Support Survey.
